# Canine Meningoencephalitis of Unknown Origin—The Search for Infectious Agents in the Cerebrospinal Fluid via Deep Sequencing

**DOI:** 10.3389/fvets.2021.645517

**Published:** 2021-12-07

**Authors:** Jasmin Nicole Nessler, Wendy Karen Jo, Albert D. M. E. Osterhaus, Martin Ludlow, Andrea Tipold

**Affiliations:** ^1^Department of Small Animal Medicine and Surgery, University of Veterinary Medicine Foundation, Hannover, Germany; ^2^Research Center for Emerging Infections and Zoonoses, University of Veterinary Medicine Foundation, Hannover, Germany

**Keywords:** dog, meningoencephalitis of unknown origin, inflammatory, brain, immune-mediated, unknown etiology

## Abstract

Meningoencephalitis of unknown origin (MUO) describes a group of meningoencephalitides in dogs with a hitherto unknown trigger. An infectious agent has been suggested as one possible trigger of MUO but has not been proven so far. A relatively new method to screen for viral RNA or DNA is next-generation sequencing (NGS) or deep sequencing. In this study, a metagenomics analysis of the virome in a sample is analyzed and scanned for known or unknown viruses. We examined fresh-frozen CSF of 6 dogs with MUO via NGS using a modified sequence-independent, single-primer amplification protocol to detect a possible infectious trigger. Analysis of sequencing reads obtained from the six CSF samples showed no evidence of a virus infection. The inability to detect a viral trigger which could be implicated in the development of MUO in the examined population of European dogs, suggests that the current techniques are not sufficiently sensitive to identify a possible virus infection, that the virus is already eliminated at the time-point of disease outbreak, the trigger might be non-infectious or that there is no external trigger responsible for initiating MUO in dogs.

## Introduction

Meningoencephalomyelitis without detectable infectious etiology is a well-known disease entity in dogs (1, 2). Several terms have been used for these inflammatory diseases of the central nervous system (CNS) (3). The most recent nomenclature uses the term “meningoencephalitis of unknown origin” (MUO) (4). Investigations of MUO cases via histopathologic examinations, immunohistochemistry (IHC), or polymerase chain reaction (PCR) have failed to provide conclusive evidence on the identity of a pathogen which may trigger the disease (5–9). Today's knowledge allows several interpretations: MUO might be a primary immune-mediated entity or alternatively a multifactorial disease in which an infectious agent or other trigger induces an inflammatory response according to the “hit and run principle” (10). This principle describes a phenomenon, in which the primary pathogen is no longer detectable, when clinical signs are recognized. Alternatively, the pathogen is novel and thus is difficult to identify using conventional techniques (5). In such circumstances, the use of advanced techniques for virus discovery may present a promising way to identify hitherto unknown infectious agents associated with MUO. Next-generation sequencing (NGS) has revolutionized the rate and breadth of virus discovery (11). This technique enables to sequence a mixture of genetic material and reveals with high sensitivity so far unknown or incomplete viral deoxyribonucleic acid (DNA) or ribonucleic acid (RNA) in a host (11). Recently NGS was used to search for candidate infectious agents in a subpopulation of North American dogs (12) with MUO without success. Viruses might have a specific geographic distribution (13) which might differ between North America and Europe.

The hypothesis should be examined that an infectious agent triggers MUO. Therefore, we report in this study a metagenomics analysis of the virome present in cerebrospinal fluid (CSF) samples from a European subpopulation of dogs with clinically suspected MUO in an acute stage of the disease or with acute relapse of clinical signs. In this early stage of the disease the possibility is enhanced that the etiological agent of this condition might be detectable. Identification of virus specifically associated with the development of MUO would greatly enhance our understanding of the pathogenesis of this disease, which could facilitate the development of a specific therapy or strategies to prevent the onset of MUO.

## Materials and Methods

### Case Evaluation and Sample Collection

Dogs included in this study were client owned patients of the Department for Small Animal Medicine and Surgery of the University of Veterinary Medicine, Hannover, Germany. All dogs were clinically investigated by at least one Resident or Diplomate of the European College of Veterinary Neurology (ECVN). Further diagnostic examinations were performed with informed written owner's consent. MRI examination (3.0 T MRI scanner Achieva, Philips Medical Systems, Best, The Netherlands) and suboccipital CSF collection was performed in general anesthesia (premedication: diazepam 0.5 mg/kg intravenously (i.v.), levomethadone with fenpipramide 0.1 mg/kg i.v. (L-Polamivet®, MSD Tiergesundheit, Unterschleißheim, Germany), induction of anesthesia: propofol dose to effect 1–4 mg/kg i.v., orotracheally intubation and connection to a semiclosed circle absorber system [Anesthesia ventilator, Cato®, Dräger, Germany), maintenance of anesthesia: isoflurane in an oxygen/air mixture (1:1, flow 50 ml/kg/min)].

### Diagnosis of MUO Cases

MUO was diagnosed based on criteria outlined by Granger et al. (4) including described clinical signs and magnetic resonance imaging (MRI) appearance as well as breed predisposition (14) and negative testing for infectious agents commonly found in the region of Northern Germany. PCR of CSF samples and serum antibodies for canine distemper virus, Toxoplasma gondii and Neospora caninum (Laboklin, Bad Kissingen, Germany) had to be negative for a case to be included within this study (4).

### Preparation of Samples for Next Generation Sequencing

CSF samples obtained from animals with suspected MUO were frozen at −80°C within 2 h after sampling and then used for NGS analysis using a modified sequence-independent, single-primer amplification protocol as described previously (15, 16). Briefly, three freeze/thaw/homogenization cycles were performed on 75–200 μl of CSF from each animal to disrupt any cells present in the samples. After centrifugation (12,000 g for 5 min at 4°C) and filtering (0.45 μm) RNA and DNA were extracted with TRIzol (Thermo Fischer Scientific, Waltham, Massachusetts, USA) and QIAamp DNA mini-Kit (Qiagen, Hilden, Germany), respectively. During the extraction of RNA from samples using TRIzol, we carefully remove the colorless upper aqueous phase which contains RNA without disturbing the interface and lower phase where DNA is present, to reduce the amount of decontamination background DNA. RNA was reverse transcribed to complementary DNA (cDNA) with a mixture of random and non-ribosomal hexamers using Superscript IV (Thermo Fischer Scientific, Waltham, Massachusetts, USA). Second-strand cDNA synthesis was performed on DNA samples and newly synthesized cDNA samples using 3′-5′ Klenow DNA polymerase (2.5U) (NEB, Ipswich, Massachusetts, USA) with the resulting dsDNA mixed at a 1:1 concentration. Random amplification of samples was performed using Taq polymerase (Thermo Fisher Scientific) as previously described (17) with only non-ribosomal hexamers. PCR products digested at 37°C for 1 h with EcoRV (NEB). The restriction enzyme was inactivated at 80°C for 20 min and digested PCR products were purified using a QIAquick PCR purification kit (Qiagen). A DNA library was constructed using the NexteraXT protocol (Illumina, San Diego, CA) with NGS performed on an Illumina MiSeq system using MiSeq Reagent kit V3 (300 × 2 cycles; Illumina, San Diego, California, USA). An additional quality control step involved exclusion of any viral sequencing reads which aligned to external samples from this study, which were processed and sequenced on the same MiSeq lane. Raw reads were screened for the presence of viral pathogens using IDseq (v3.5) Portal (https://idseq.net), a cloud-based, open-source bioinformatics platform designed for rapid identification of pathogens in metagenomics data, as described previously (18, 19). In brief, host reads, duplicates and lowquality reads are excluded via an algorithm. Non-host reads are than aligned to NCBI nucleotide and protein database (18, 19).

## Results

Six dogs with clinically suspected MUO were included in this study (Table 1). Diagnosis of MUO was made based on standard criteria: Clinical signs were in accordance with an intracranial focal to multifocal lesion. MRI showed multifocal intraaxial lesions with no to minimal mass effect in the white and/or gray matter of the cerebrum, cerebellum and/or brainstem (6/6). In most dogs (5/6) minimal to moderate contrast enhancement was seen. In 4/6 dog's CSF pathological changes as increased cell count and/or protein content were visible (Table 2). Results implied an inflammatory brain disease. Testing for standard infectious agents were negative. Raw sequencing reads obtained from NGS performed on RNA and DNA samples extracted from six CSF samples were analyzed using IDseq. The number of reads which passed host filtering and quality control ranged from 336,931 to 1,640,976 (Table 2). An additional quality control step involved exclusion of any viral sequencing reads which aligned to external samples from this study which were processed and sequenced on the same MiSeq lane. Upon completion of the quality control steps, no sequencing reads specific to viral pathogens known to infect mammals could be detected in the MUO CSF samples (Table 2).

**Table 1:** Clinical details of canine patients with clinical suspected meningoencephalitis of unknown origin.

**No**.	**Breed**	Age (years)^*^	**Gender**	**Duration of clinical signs before presentation**	**Neuro-localization**	**MRI**	**CSF**	
							**Cell count (/3μl^#^) differentiation**	**Protein (mg/dl)**
1	Small munsterlander	2	fn	Progressive over 3 months	Forebrain	Multifocal subcortical white matter, contrast+	28 mononuclear	127
2	Airedale terrier	5	fn	Progressive over 1 week	Forebrain	Multifocal subcortical white matter, contrast+	0	49
3	Yorkshire terrier	3	mn	Progressive over 2 weeks	Multifocal intracranial	Multifocal subcortical white matter, contrast+	8 mixed	54
4	Yorkshire terrier	9	f	Progressive over 2 weeks	Multifocal intracranial	Multifocal subcortical white matter, contrast+	5 Mononuclear	48
5	Biewer yorkshire terrier	2	mn	Progressive over 2 weeks	Multifocal intracranial	Subcortical white matter, brainstem, cerebellum, contrast+	1 Np	13
6	Yorkshire terrier	5	m	Chronic over since 2 years, acute deterioration	Multifocal intracranial	Multifocal cortical gray matter, thalamus, generalized brain atrophy, contrast-	4 Np	14

* *Rounded*;

# *counted with Fuchs-Rosenthal-chamber; mn, male neutered; fn, female neutered; f, female; m, male; MRI, magnetic resonance imaging; contrast+ contrast enhancing lesions; contrast-, non-contrast enhancing lesions; CSF, cerebrospinal fluid; mixed, mixed cell population of neutrophils and mononuclear cells; np, cell differentiation was not performed*.

**Table 2:** Overview of samples used for next generation sequencing analysis.

**No**.	**CSF volume (μl)**	**DNA yield (ng/μl)**	**No. of total reads**	**Passed QC^1^ (%)**	**No. of reads^2^**	**Viruses detected^3^**
1	150	0.048813	2,741,706	75.98	1,640,976	· Propionibacterium virus ATCC29399BC (442 reads) · Pepino mosaic virus (116 reads)
2	250	0.053985	2,135,708	66.87	574,390	· Carrot cryptic virus (14 reads)
3	250	0.047843	2,778,556	72.05	871,482	· Pa6virus (550 reads) · Streptococcus phage IPP16 (76 reads) · Spodoptera frugiperda granulovirus (2 reads)
4	75	0.049244	1,458,200	55.37	336,931	–
5	75	0.048705	2,244,666	65.29	534,895	· Acinetobacter phage Acj61 (214 reads)
6	200	0.049136	2,189,290	75.29	584,093	· Acinetobacter phage Acj61 (210 reads)

1 *passed quality control (QC) percentage represents the proportion of reads that passed sequence quality thresholds*;

2 *the number of original sequencing reads that are sent to downstream analysis after host and quality filtering*;

3 *only reads with an alignment length ≥30bp are shown*.

## Discussion

MUO is an umbrella term describing inflammatory changes of the CNS with suspected non-infectious etiology (4). Thus, far, no infectious agents have been detected as a potential trigger for the exacerbating immune response using histopathological or immunohistochemical techniques, virus isolation or PCR (5–9). The dilemma is that absence of evidence is not evidence of absence. Immunohistochemistry or PCR can only detect pathogens, where a specific knowledge about the presumed pathogen is present (20). In the current study, a novel non-specific viral detection technique was applied, which was already successfully used for metagenomic investigations in human CSF (21–23) and various veterinary samples (24–26): Sensitivity and specificity of NGS in human CSF sample was 95 and 96%, respectively (21).

NGS sequencing discovers viral DNA or RNA without requiring any prior knowledge (27) of specific viruses. With this technique, for example Batai Virus Encephalitis in Harbor Seals was detected in brain parenchyma (25). In dogs with MUO an US-American research group examined fresh samples of brain parenchyma and CSF of affected and unaffected control dogs as well as various positive control samples with NGS. They detected occasional DNA or RNA of Pseudomonas, Streptococcus, Staphylococcus species and bacteriophages, but no consistent and specific candidate in cases of MUO (25). Here, no specification about chronicity of MUO was made (12).

One of the major dilemmas is the diagnosis of MUO. MUO includes several subtypes of meningoencephalomyelitis: granulomatous meningoencephalomyelitis (GME), necrotizing leukencephalitis (NLE) and necrotizing meningoencephalitis (NME) are the subtypes most frequently described (2–6). Although these entities clinically can often be distinguished by typically affected breed, age of onset and affected brain area as well as appearance in MRI and CSF cell count, some research suggests, that overlaps might exist (2, 4, 14, 28). Therefore, some authors suggests to use the term MUO if no histopathological examination was performed (4). In addition, the clinical diagnosis of MUO is a diagnosis of exclusion, when the following results are found: (a) the clinical examination suggests a focal or multifocal intracranial lesion; (b) diagnostic imaging reveals a multifocal to diffuse intra-axial lesion, preferably with contrast enhancement; (c) pleocytosis and increased protein is evident in the CSF and (d) endemic infectious diseases are excluded (2–9, 14). Nevertheless, normal CSF findings are possible in dogs with MUO (4). According to the current state of knowledge, histopathology after brain biopsy is necessary to finally confirm the disease. Without such a diagnostic tool MUO remains a presumptive diagnosis (4). However, brain biopsy is highly invasive and often permission of the patient's owner is missing.

Distinct triggers might be involved in the pathogenesis of the different MUO subtypes. Searching such a trigger in a homogenous population of MUO subtypes might be helpful, but was not successful as previously published (2, 4, 8). Therefore, the present study used a different approach. The goal was to pilot a search method for infectious agents screening CSF samples of several subtypes of MUO based on clinical presumptive diagnosis to depict heterogeneous presentation of MUO in a clinical setting. Using this heterogeneous group of dogs should increase the likelihood to find an infectious trigger in one of the subtypes. In case of positive findings results could have been confirmed by sampling probes from a homogenous population evaluating one subtype of MUO. The CSF samples were taken at an acute stage of the disease or at the time point of acute relapse of clinical signs, where the odds are high, that the infectious agent might still be detectable.

In the CSF samples, a high percentage of non-host reads were found. It is known that in clinical samples such as CSF which contain very little biomass, the primers over-amplify very small quantities of contaminants and therefore increase the proportion of irrelevant contaminates in the final dataset (29). To reduce background contamination pretreatment with deoxyribonuclease might be used (30). But this might reduce the chance to find DNA virus sequences (30). Therefore, the present study used other methods to decrease background contamination that incorporated the use of non-ribosomal hexamers (16) and specific sample processing technique. Bioinformatic processing was performed via IDseq, which represents a new gold standard for optimized screening of NGS data for known and unknown viral pathogens (18, 19). IDseq has been used previously to successfully identified pathogens in CSF samples (21). Of the sequencing reads with an alignment length ≥30bp, only DNA or RNA of bacteriophages or plant or insect viruses were identified in the current study (31–37). Although a connection between unspecific immune response in humans with positive stool samples for a pepper associated virus has been suggested (38), it is largely presumed that plant and insect viruses do not cause clinical signs in mammals (39). Therefor the viruses found in the CSF samples are interpreted as background contamination and are hence considered clinically irrelevant (40). Therefore, no evidence of novel or known viral pathogens with the potential to infect mammals was found following analysis of sequencing reads in this study obtained from samples of European dogs. This complements a previous metagenomics study that also failed to identify possible triggering pathogens in samples obtained from North American dogs with MUO (12).

Although the CSF samples in the current study did not contain viral RNA or DNA which could be associated with MUO, negative NGS results cannot exclude an infectious etiology. An infectious pathogen, which was potentially involved in initiating the inflammation, could have been largely eliminated by the immune system without impeding the inflammatory host response [“hit and run”-theory (9, 41)]. NGS of samples from dogs with acute or renewed flare-up of inflammation might still offer the best chance to detect the pathogen, before it is eliminated by the immune system. The evaluation of CSF samples using NGS increases the chances of detecting any RNA or DNA of a candidate virus associated with MUO. In human medicine search for DNA of infectious agents in CSF via NGS has a sensitivity of 73% and a specificity of 99% (42), but the optimal protocol for NGS is dependent on the pathogen (43). Therefor the diagnostic yield might be lower for certain viruses with the protocol described herein.

Summarizing, we could not confirm the hypothesis that MUO might be caused by an infectious trigger. Nevertheless, our current state of knowledge of MUO is suggestive of a multifactorial etiology, including an underlying genetic susceptibility and involving an additional unknown external trigger (44). The possibility remains that the trigger might not be an infectious agent but another environmental noxa. In several dog breeds, such as Pug Dogs, Maltese, and Chihuahua, a genetic defect in DLA-II is known to increase the risk of developing MUO (45, 46). The use of novel technologies used to identify the etiology of possible pathogens associated with neurological disease in humans via identification of virus-specific antibodies in CSF, may also be of value in the field of veterinary medicine in investigations of disease syndromes such as MUO. The search for the origins of MUO will in future require increased cross-disciplinary investigations to determine the contributions of a postulated pathogen and the host immune response in determining the pathogenesis of this disease.

## Data Availability Statement

The datasets presented in this study can be found in online repositories. The names of the repository/repositories and accession number(s) can be found below: https://www.ncbi.nlm.nih.gov/sra/PRJNA698629.

## Author Contributions

JN collected clinical data, examined cases, collected CSF samples, wrote, and finalized the report. WJ analyzed CSF samples and NGS data. AO designed the study and finalized the report. ML analyzed the NGS data, supervised the CSF analysis, and wrote the report. AT designed the study, collected CSF samples, and finalized the report. All authors contributed to the article and approved the submitted version.

## Funding

This study was supported by a grant from the Niedersachsen-Research Network on Neuroinfectiology (N-RENNT) from the Ministry of Science and Culture of Lower Saxony, Germany, and by Deutsche Forschungsgemeinschaft and University of Veterinary Medicine Hannover, Foundation within the funding programme Open Access Publishing.

## Conflict of Interest

The authors declare that the research was conducted in the absence of any commercial or financial relationships that could be construed as a potential conflict of interest.

## Publisher's Note

All claims expressed in this article are solely those of the authors and do not necessarily represent those of their affiliated organizations, or those of the publisher, the editors and the reviewers. Any product that may be evaluated in this article, or claim that may be made by its manufacturer, is not guaranteed or endorsed by the publisher.
